# A High-Throughput Automation Platform for Accelerated AAV Stability Optimization

**DOI:** 10.3390/pharmaceutics18050608

**Published:** 2026-05-16

**Authors:** Shuai Li, Xiaoyan Wang, Li Zhi, Mohammed Shameem, Dingjiang Liu

**Affiliations:** Drug Product Development and Technology, Regeneron Pharmaceuticals Inc., Tarrytown, NY 10591, USA; shuai.li@regeneron.com (S.L.); xiaoyan.wang@regeneron.com (X.W.); mohammed.shameem@regeneron.com (M.S.)

**Keywords:** AAV, formulation, high-throughput screening, automation

## Abstract

**Background**/**Objectives:** Recombinant adeno-associated virus (AAV) stands at the forefront of gene therapy development, requiring stable formulations to support the expanding therapeutic applications. The growing diversity of serotypes and engineered capsids often creates complex challenges for formulation development, thus demanding innovative formulation development strategies beyond traditional manual approaches to characterize a large formulation design space quickly to discover stable formulations. **Methods:** Here, we address this critical need through a high-throughput automation platform that dramatically enhances formulation development efficiency and capability through rapid formulation preparation and high-throughput AAV analytics. This system prepares 96 distinct formulations in 40 min and completes AAV compounding in 20 min per plate, with precise control of pH, buffer components, and AAV titers. **Results:** In a proof-of-concept formulation development study using AAV1, we screened 128 formulations across multiple buffer systems, pH ranges, and excipient combinations. This comprehensive approach successfully identified optimal stable high-titer AAV1 formulations (1.2 × 10^14^ vector genome (vg)/mL) that maintained stability under frozen, refrigerated, and room temperature storage conditions. **Conclusions:** Our study demonstrated that this automation platform combined with high-throughput AAV analytics significantly accelerates formulation development, conserves AAV material, and enables systematic exploration of broader formulation design space. It allows us to achieve identification of robust and stable AAV formulations within a timeframe unmatched by traditional formulation development approaches.

## 1. Introduction

The recombinant adeno-associated virus (AAV) vectors are non-pathogenic, replication-deficient viruses that can deliver therapeutic genes to target cells [1,2,3]. The clinical success of AAV-based therapies, exemplified by eight commercially approved products since 2017, has accelerated development in the AAV gene therapy field, offering a promising avenue for the treatment of a diverse array of genetic diseases [4,5]. This expanding scope covers multiple serotypes, engineered capsids, and therapeutic payloads, each requiring optimized formulations to ensure stability, safety, and clinical efficacy [6,7].

Formulation development represents a critical yet often underappreciated challenge in advancing AAV therapeutics from discovery to clinical application. Although commonly used frozen liquid AAV formulations are often sufficiently stable for many naturally occurring AAV serotypes [8,9], stability issues may arise for certain AAV serotypes with inherent conformational instability (e.g., AAV1, AAV2), high-titer AAV formulations, and AAVs using engineered capsids. Under these circumstances, stability concerns must be addressed through systematic optimization of buffer components, pH conditions, and excipient combinations [7,8,10,11,12]. Traditional approaches to formulation development rely heavily on labor-intensive manual compounding and screening methods that severely constrain the capacity for exploring formulation design space necessary for identifying optimal conditions. The manual preparation of buffer solutions often involves pipetting, weighing, and mixing of individual excipient components. This approach is not only prone to human error but also limits the number of buffer variants that can be practically prepared and assessed within a given timeframe. Recently, this limitation becomes particularly problematic with the anticipated increase in the number of different AAV serotypes, engineered capsids, and the development of retargeted AAV technology [4,13,14,15]. These capsids may exhibit distinct physicochemical properties and stability profiles compared to well-characterized nature variants. Moreover, there is a growing demand for developing stable AAV formulations suitable for higher titers (i.e., >1.0 × 10^14^ vg/mL) and adaptable for non-frozen storage conditions at 2–8 °C [16,17,18]. The conventional AAV formulations developed for deep frozen storage are no longer sufficient to meet these evolving requirements [17]. Consequently, there is a pressing need to develop a high-throughput (HTP) formulation development platform to enable exploring a much broader formulation design space. Such capabilities are particularly valuable given the accelerating pace of gene therapy development and the growing emphasis on platform approaches that can accommodate diverse AAV serotypes and capsids, titers and different transgene sizes [16,19].

To achieve efficient HTP formulation development, there are a few technical hurdles that must be overcome: (1) precise compounding of AAV material to maintain consistent AAV concentration across numerous test conditions; (2) an appropriate plate-based container which demonstrates good material compatibility with AAV and enables low-volume formulation screening; (3) a reliable plate-sealing method which minimizes compounding variability due to evaporation and edge effects; (4) HTP analytical instruments which demonstrate satisfactory and comparable assay performance as conventional analytical methods. Effectively addressing these technical hurdles is essential for establishing the HTP approach for rapid optimization of formulations tailored to specific AAV constructs.

Recently, automation technology has appeared as a transformative solution to these constraints by enabling HTP, precise, and reproducible formulation screening [20,21,22,23,24]. Firstly, automated liquid handling systems can accurately and rapidly dispense precise volumes of buffer components, significantly reducing the time and effort required for buffer preparation [22,25,26]. Moreover, HTP analytical techniques can also aid in providing rapid and detailed characterization in different buffer conditions [27]. While automated approaches have revolutionized various aspects of biopharmaceutical development, their application to AAV formulation development remains largely unexplored [28,29,30,31]. The integration of liquid handling systems, storage condition environment controls, and analytical methods presents an opportunity to dramatically expand the formulation design space, identifying the best formulation “hits” through the screening process while reducing development timelines critical for early-stage clinical development. This data-rich and systematic approach also helps to identify the pH and excipient concentration ranges that support AAV stability, which are valuable for formulation robustness studies required for late-stage clinical manufacturing. Recent advancements in HTP AAV analytical techniques facilitate this process. For instance, the Stunner system (Unchained Labs, Pleasanton, CA, USA) assesses the concentration and particle size, the Wyatt plate reader (Waters, Milford, MA, USA) dynamic light scattering (DLS) detects aggregation, the Halo Labs system (Waters) analyzes sub-visible particulates, and the liquid chromatography (LC) system quantifies released genome DNA concentration [32]. These methods enable simultaneous evaluation of multiple stability-indicating critical quality attributes (CQAs) across all formulation conditions, generating rich datasets that capture the multifaceted nature of AAV stability.

Here, we present a comprehensive automation platform for AAV formulation development that addresses these unmet needs. Our protocol enables rapid preparation of diverse buffer systems, precise compounding, and reliable stability assessment across 96-well plate formats. Through systematic optimization of liquid handling parameters, we achieved exceptional precision in controlling buffer pH, excipient concentration, and AAV titer across the 96-well plate. By implementing a robust sealing method, the 96-well plate-based storage conditions showed no evaporation or edge effect issues. In addition, the platform, combined with advanced HTP analytical techniques and data analysis, was verified with a case study for AAV1 formulation screening of 128 formulations across multiple buffer systems, pH ranges, and excipient combinations. A similar amount of formulation preparation work that could take about one week for an analyst to complete manually can now be accomplished within approximately two hours, taking advantage of this high-throughput automation platform. The results from this verification study successfully proved the capability of the HTP automation platform for identifying top-performing stable AAV formulations.

Overall, by streamlining the processes of buffer preparation, screening, and characterization, this automation-assisted HTP AAV formulation development platform holds great promise for the development of stable high-titer AAV formulations and contributes to the advancement of gene therapy as a viable treatment option for genetic diseases.

## 2. Materials and Methods

### 2.1. Materials

AAV materials were produced in-house at Regeneron Pharmaceuticals, Inc. (Tarrytown, NY, USA) using a triple-transfection process in HEK293 cells (Thermo Fisher, Waltham, MA, USA). The downstream purification process included affinity chromatography, followed by anion-exchange chromatography for the enrichment of full capsids. The purified AAV materials were characterized by baseline purity and quality attributes prior to formulation studies and showed approximately 80% full capsids without apparent aggregates. Buffer components including Tris Base, Tris Acid, Phosphate Monobasic, Phosphate Dibasic, Sodium Acetate, Acetic Acid, and Mannitol, Arginine Hydrochloride (ArgHCl), and Sodium Chloride (NaCl) were purchased from J.T. Baker, Avantor (Radnor, PA, USA). Poloxamer 188 (P188) was purchased from BASF (Ludwigshafen, Germany). Sucrose was purchased from Ferro Pfanstiehl (Waukegan, IL, USA). Food red dye was purchased from TCI America (Portland, OR, USA).

### 2.2. Automated Buffer Preparation and Compounding

**Preparation Work:** All necessary materials, including reagents, consumables, high-concentration stock solutions of buffers, stabilizers, surfactants, and AAV samples were prepared before starting automation runs. A comprehensive formulation preparation list was created using Excel (Supplemental Document S1), which included target pH, excipient types, and concentrations of stock solutions. The system automatically calculated the exact volume of stock solutions to be transferred to each 96-well plate. The original excipient concentration in AAV concentrate and the dilution factor of AAV during compounding were incorporated into calculations to ensure formulation accuracy. **Buffer Preparation:** The Hamilton STAR liquid handling system (Hamilton, Reno, NV, USA) was configured to prepare buffer mixtures in a 2 mL Axygen 96-well plate (Corning Axygen, Union City, CA, USA). An input file based on the calculations from the Excel file (Supplemental Document S1) was created to specify the exact transfer volume of each stock solution. The automated system used 1 mL hanging sterile conductive filter tips (Hamilton) to dispense precise volumes of stock solutions of Tris Base, Tris Acid, Phosphate Monobasic, Phosphate Dibasic, Sodium Acetate, Acetic Acid, surfactants, and other excipients into designated wells on the 96-well plate, creating a comprehensive buffer library for subsequent screening. **Sample Transfer (Compounding):** Prior to compounding, AAV samples were concentrated to approximately the target titer using Amicon ultra centrifugal filters with 30 kDa Molecular Weight Cutoff (MWCO) (MilliporeSigma, Burlington, MA, USA). A total of 135 µL AAV concentrate was added to each well in the first two columns of a Bio-Rad 96-well PCR plate (Bio-Rad, Hercules, CA, USA). The Hamilton STAR liquid handling system mixed the buffers in the 2 mL Axygen 96-well plate using a 96-tip stamp head pipette system and 300 µL hanging sterile conductive filter tips (Hamilton) to ensure buffer homogeneity in the well. Subsequently, 180 µL of the prepared buffer was transferred column by column to a Crystal Zenith^®^ (CZ) 96-well plate (Daikyo Seiko, Tochigi, Japan) using an 8-tip channel pipette. Then 20 µL of AAV concentrate from each well of column 1 of the Bio-Rad plate was transferred to column 1 of the CZ plate containing 180 µL target formulation buffers per well. This transfer process was repeated for an additional 5 times until columns 1–6 of the CZ plate were filled. Similarly, 20 µL of AAV concentrate from each well of column 2 of the Bio-Rad PCR plate was transferred to columns 7–12 of the CZ plate. The CZ plate containing formulated AAV samples was covered with FluoroTec-coated butyl rubber 96-well plate mats (Daikyo Seiko), sealed with Parafilm sealing film, and placed in a sealed Mylar bag. The prepared plate was then transferred to an incubator set at the target temperature.

### 2.3. Multi-Attribute Analysis by Stunner

Vector genome titer and particle size distribution were quantified using Stunner (Unchained Labs). This system integrates UV-Vis spectrophotometry, static light scattering (SLS) and DLS for AAV characterization. Then, 2 µL of each AAV sample was loaded onto a Stunner Plate (Unchained Labs) using water as blank and characterized with the “AAV Quant” application using default (4 × 5 s) DLS acquisition settings at room temperature. The nucleic acid sequence of the single-stranded DNA (ssDNA) transgene in the AAV samples and the amino acid sequences of capsid viral proteins were used to calculate the vector genome titer results. Data analysis was automatically performed using Lunatic & Stunner Analysis Software (version 8.2.0.259, Unchained Labs).

### 2.4. Particle Size Distribution by DLS

Particle size distribution was determined using the Zetasizer Ultra system (Malvern Panalytical, Malvern, UK). First, 45 µL of each AAV sample was loaded onto a ZEN2112 low-volume quartz cuvette (Malvern Panalytical) and equilibrated at 25 °C for 180 s in the instrument prior to measurement. AAV samples were measured at a 90° angle. Five replicate measurements were collected with 10 acquisitions, each for 10 s. Data collection and analysis were performed with ZS Xplorer software (version 3.3.0.42, Malvern Panalytical).

### 2.5. Vector Genome Titer by Droplet Digital PCR (ddPCR)

Vector genome titer was determined using the QX200 ddPCR system (Bio-Rad Laboratories). AAV samples were treated with DNase I (Thermo Fisher) to eliminate unencapsulated DNA, followed by Proteinase K (Thermo Fisher) digestion. Serial dilution to a concentration range of 200 to 8000 copies/µL was performed for the digested AAV samples with dilution buffer (20 mM tris, 0.05% (*v*/*v*) P188, 0.002 µg/µL sheared salmon sperm DNA, pH 8.0). The samples were mixed with ddPCR master mix (Bio-Rad) and transgene specific primer probe set and then partitioned on an Automated Droplet Generator (Bio-Rad). After the droplet generation, PCR amplification was performed using a thermal cycler (Bio-Rad). Following PCR amplification, AAV vector genome titer was calculated with QxManager Software (version 1.2.345.0909, Bio-Rad) on a QX200 Droplet Reader (Bio-Rad).

## 3. Results

### 3.1. Description of the Workflow

The workflow of the automated, low-volume, 96-well plate-based formulation screening platform is illustrated in graphical abstract. The workflow comprises four sequential processes: (1) formulation buffer preparation, (2) AAV compounding, (3) HTP analytical testing, and (4) stability confirmation study and data analysis.

#### 3.1.1. Formulation Buffer Preparation

The workflow begins with the rational design of formulation matrices that systematically explore the multidimensional formulation space of buffering agents, pH conditions, and excipient combinations. A template Excel spreadsheet (Supplemental Document S1) is used for calculating the volumes required for each excipient stock solution prepared ahead to achieve the target excipient concentration in the designed formulation buffers. These calculated volumes are subsequently input into the Hamilton Microlab STAR (or other similar) automated liquid handler system. This system is programmed to prepare formulations in a 96-well plate format.

#### 3.1.2. AAV Compounding

Following formulation buffer preparation, this platform performs automated addition of high-concentration AAV stock to each formulation buffer. CZ 96-well plates are utilized for formulation screening to align with the primary container material used in most of the currently marketed AAV drug products. After compounding, the plates undergo a multi-layer sealing process to eliminate evaporation and edge effects during extended storage, and are then transferred to controlled-temperature storage units for predetermined time intervals, with typical stability studies examining multiple time points across relevant temperature conditions.

#### 3.1.3. HTP Analytical Testing

At each time point, the platform performs automated sampling with minimal volume for characterization of all formulations using plate reader-based HTP analytical methods including vector genome titer quantification and particle size distribution analysis by Stunner. Upon completion of analysis, data are summarized and visualized to identify optimal formulations.

#### 3.1.4. Stability Confirmation Study and Data Analysis

The promising formulations identified based on the screening results undergo stability confirmation studies using AAV drug product configuration and increased titer to verify formulation performance and assess formulation robustness at high titer. Advanced statistical analysis can be further performed to elucidate the relationship between formulation parameters and product quality attributes, facilitating the identification of formulation design spaces that maintain satisfactory stability. If the identified formulations fail to meet the requirements for AAV stability during the confirmation study, the data collected will be used to improve the rational design of the formulation matrices to further optimize the AAV formulations. This iterative process ensures identification of stable and robust formulations sufficient for supporting the long-term storage, manufacturing process, handling, and clinical administration of AAV drug products.

### 3.2. Development of the HTP Formulation Screening Platform

To achieve precise formulation preparation, AAV compounding and formulation screening process, several key steps in the automation protocol were optimized by introducing optimized liquid handling parameters, instantaneous preparation of AAV concentrate stock solutions, minimal-waste transfer techniques, and optimized multi-layer sealing strategy to prevent plate evaporation.

#### 3.2.1. Optimization of Automated Liquid Handling Parameters for Preparing Formulation Buffers

To begin, the automated liquid handling parameters were systematically optimized to ensure accurate transfer of diverse components with varying physicochemical properties. The liquid classes were customized for the automated handler to account for the specific flow characteristics of high-viscosity components. In addition, tip positioning under the buffer surface during sample addition was precisely controlled to minimize splashing and ensure quantitative transfer. This step is especially critical for surfactant-containing formulations due to a propensity for bubble formation during the liquid handling process on the pipette tips. The mixing protocol following sample addition was calibrated to ensure good homogeneity while minimizing the introduction of air bubbles.

**Figure 1:**
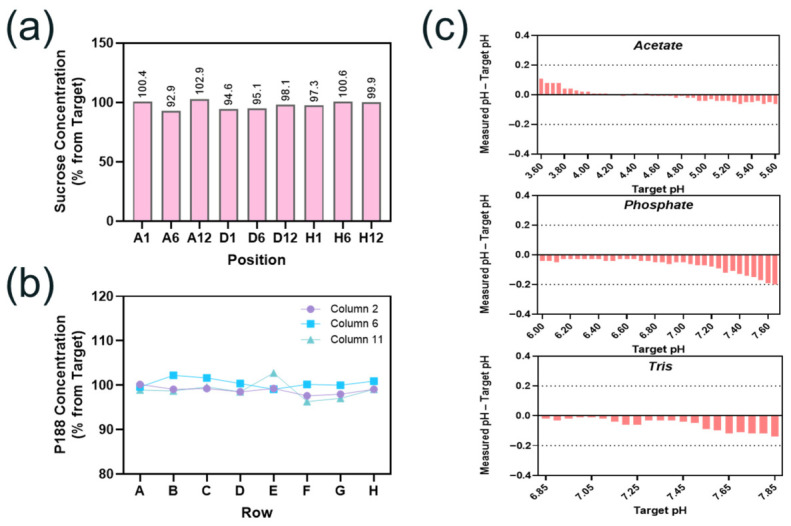
Validation of automated formulation preparation process. Samples prepared using the automated formulation process showed comparable sucrose concentrations (**a**); Poloxamer 188 (P188) concentrations (**b**); and pH (**c**) as the target values.

The platform’s capability for precisely compounding excipient components was assessed using sucrose as a cryoprotectant and P188 as a surfactant. We programmed the system to prepare formulations with the target concentration of either 1.5% *w/v* sucrose or 0.0054% *w/v* P188 across a full 96-well plate. The sucrose concentration of nine representative samples from the 96-well plate (positions A1, A6, A12, D1, D6, D12, H1, H6, and H12 to cover corner, edge, and center positions across the plate) was measured using a reversed-phase ultra-high-performance liquid chromatography (RP-UPLC) method, and the data are shown in Figure 1a. We found that the difference between the measured and the target sucrose concentrations was less than 8% with a coefficient of variation (CV) of 3.3% among all the samples measured. Similarly, the concentration of P188 of 24 representative samples across the 96-well plate (columns 2, 6, and 11 representing left, center, and right regions of the plate) was measured using a size exclusion chromatography with charged aerosol detector (SEC-CAD) method. As shown in Figure 1b, the difference between the measured and the target P188 concentrations was within 4% with a CV of 1.5% among the 24 samples analyzed. These results demonstrate that the optimized automated liquid handling platform is able to prepare formulation buffers with consistent and accurate excipient levels across 96-well plates.

#### 3.2.2. pH Control via Pre-Adjustment

pH is a critical formulation parameter. The platform’s pH control capability was assessed using three buffer systems commonly employed in biological product formulations: acetate (pH 3.6–5.6), phosphate (pH 6.0–7.65), and tris (pH 6.85–7.85). The automated liquid handler created pH gradients with 0.05 unit increments between adjacent wells by dispensing precisely calculated volumes of acid and conjugate base components based on the Henderson–Hasselbalch equation [33]. We found that pre-adjustment of the calculated volume ratio of the acid and base stock solutions for certain buffers was necessary under our experimental conditions to compensate for the apparent pKa shift caused by the ionic strength contribution from formulation excipients. When ionic excipients such as NaCl and Arginine HCl are added to formulation buffers, the activity coefficients of charged buffer species may be altered due to the ionic strength increase, thereby shifting the apparent pKa away from the thermodynamic pKa value [34,35]. The magnitude of the required pre-adjustment was found to be different for the three buffer systems used in this study because of their distinct ionic strength sensitivity. The specific pre-adjustment offsets required to achieve target pH were determined based on the excipient composition of the buffer system and optimized experimentally for each buffer. By applying these optimized offsets, we were able to achieve good pH accuracy across the entire pH range evaluated in this HTP formulation screening study, with 84% of the 96 formulations achieving within 0.1 units and 100% within 0.2 units of the target pH values (Figure 1c). Each of the three buffer systems assessed exhibited excellent linearity between the calculated and measured pH values (R^2^ > 0.99).

#### 3.2.3. Overcoming Material Limitations for AAV

During the early-stage formulation screening study, material availability usually is a limiting factor which can be especially serious for AAV due to the typically low production yield. To conserve the precious AAV materials, Bio-Rad Hardshell PCR plates were selected for the HTP screening study due to their minimal dead volume characteristics allowing us to reduce the dead volume waste. In addition, an optimized AAV transfer method was developed to further reduce material wastage during AAV compounding. Traditionally, automated formulation compounding workflow often adopts “plate-to-plate” transfer for improved efficiency. Following this approach, the AAV concentrate stock solution is dispensed to all the 96 wells on the PCR plate. Then, a 96-tip stamp head pipetting system capable of pipetting all 96 wells at once is used to transfer the AAV concentrate stock from the PCR plate to the 96 wells on the receiving CZ plate containing formulation buffers for screening. Although this approach streamlines the 96-well plate applications, material wastage could become a limiting factor for AAV as each of the 96 wells on the PCR plate requires approximately 15 µL dead volume for accurate liquid transfer. To overcome this challenge, a pooled AAV transfer protocol was developed where AAV concentrate stock solutions were only dispensed into the first two columns on the PCR plate and subsequently transferred to the 96 wells on the receiving CZ plate using the eight-tip channel pipette. This method, though not as efficient as the direct “plate-to-plate” transfer approach, substantially reduces material wastage due to dead volume by approximately 83% (from 1.44 mL to 0.24 mL).

#### 3.2.4. Handling High-Titer AAV Stock Solution

Another critical challenge in HTP AAV formulation screening is the requirement of concentrated AAV stock solutions which are prone to stability issues such as aggregation and formation of particulates. This can lead to heterogeneous aliquots of the AAV concentrate solution being transferred during AAV compounding, resulting in inaccurate AAV concentrations in the formulations for screening. To mitigate this issue, AAV samples were concentrated to high titer immediately before addition to formulation buffers. By minimizing the time that AAV remains at high titer, it helps to preserve vector quality and prevent development of instability issues. The process was integrated into the automated workflow to maintain consistency of AAV compounding across all formulation conditions.

**Figure 2:**
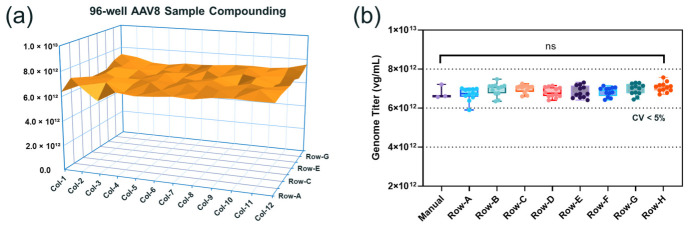
Validation of automated recombinant adeno-associated virus (AAV) compounding process. (**a**) Vector genome (vg) titer distribution across the 96-well plate prepared by compounding AAV8 concentrate stock solution (7 × 10^13^ vg/mL) with 10-fold dilution in AAV buffer; (**b**) row-wise distribution analysis showing coefficient of variation (CV) within 5% across rows A–H (n = 12 wells per row). Statistical comparison (one-way ANOVA) showed no significant difference between manually prepared samples (n = 3) with any row of samples prepared with automation platform. ns (not significant): *p* > 0.05.

To evaluate the performance of AAV compounding following the optimized protocol described above, AAV8 was concentrated to approximately 7 × 10^13^ vg/mL using a 15 mL Amicon ultra centrifugal filter with 30 kDa MWCO immediately prior to 10-fold dilution into the same AAV8 formulation buffer described previously across a 96-well plate. Vector genome titer analysis performed using Stunner showed consistent vector genome titer across all 96 samples with an overall CV of 4.2% (Figure 2a). Since the eight-tip channel pipette was utilized for the AAV compounding step, an analysis of consistency in AAV titer after compounding across the eight rows A-H was performed, which revealed CV values within 5% across rows A-H (Figure 2b). Importantly, statistical analysis showed no significant differences between automated and manually prepared AAV8 samples with the same target titer. While this does not prove formal statistical equivalence, the consistency in the measured titer values across the plate as well as the comparable measurement precision from samples prepared using either automated or manual approaches can support the practical equivalence of the two sample preparation methods for formulation screening. We understand that a formal equivalence test such as the two one-sided tests (TOST) procedure could further strengthen this comparison. It could be applied to future validation studies when a pre-specified equivalence margin can be established. Capsid titer analysis by size exclusion chromatography with multi-angle light scattering (SEC-MALS) also showed comparable results (Figure S1). This suggests the automated AAV compounding process can achieve excellent accuracy and precision which are comparable to the level achieved by skilled analysts. Furthermore, this automated process mitigates the risks with analyst-dependent variability and human errors, and meanwhile significantly improves process efficiency and productivity.

#### 3.2.5. Mitigating Evaporation and Edge Effects Associated with 96-Well Plates

Finaly, HTP screening protocols employing 96-well plates present two major challenges: evaporation and edge effects [36,37,38]. These phenomena can lead to variable levels of increases in AAV and excipient concentrations in formulations across the 96-well plate, thus skewing analytical results and data interpretation during formulation screening studies. To overcome these challenges, a multi-layer sealing strategy was developed which uses FluoroTec-coated butyl rubber mats, parafilm overlay, and mylar bag enclosure prior to exposing the plates to various assessment conditions. This ensures accurate formulation conditions across the 96-well plate can be achieved during extended incubation, especially under elevated temperatures.

**Figure 3:**
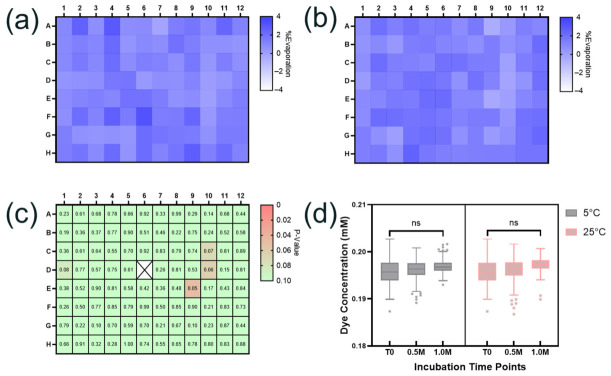
Evaluation of evaporation and edge effect on 96-well plate. Average percent evaporation based on changes in food dye sample concentrations after one month’s incubation at 5 °C (**a**) and 25 °C (**b**) (n = 3 for each sample). (**c**) Dunnett’s post hoc test performed on the plate incubated at 25 °C shows statistical differences for percent evaporation as a function of position, compared to the center well (marked as X). *p* values were calculated using Dunnett’s test in R. (**d**) Food dye concentration remained stable over one month’s incubation at 5 °C and 25 °C. Welch’s *t*-test was performed to compare the concentrations measured between the two time points (T0 vs. 1 month) using three replicate plates at each temperature (n = 288 for each time point). ns (not significant): *p* > 0.05.

The effectiveness of the multi-layer sealing strategy was assessed by adding 200 µL of 0.2 mM red food dye to each well on a 96-well CZ plate and incubating the plate at 5 °C and 25 °C for up to one month. Triplicate plates were prepared for each temperature condition. Upon completion of the incubation, concentrations of the food dye in each well were analyzed by measuring absorbance at 506 nm (A506) using the SpectraMax 190 Microplate Reader (Molecular Devices, San Jose, CA, USA). Since changes in sample volumes in the well due to evaporation would show a linear correlation with changes in A506 (Figure S2), %Evaporation can be calculated based on changes in the A506 in each well before and after the incubation. As illustrated in Figure 3a,b, the maximum %Evaporation observed at 5 °C and 25 °C after one month across the entire plate was within 3%. Statistical analysis comparing each well and the center well (well 6D) showed *p* ≥ 0.05 values for all the wells at 25 °C after one month (Figure 3c). Furthermore, no significant changes in the food dye concentrations in the 96 samples were observed with increased incubation time (Figure 3d). These results clearly demonstrate the effectiveness of the multi-layer sealing protocol in mitigating evaporation across the 96-well plate, thus minimizing position-dependent variability and edge effect, which is critical for ensuring sample integrity and the successful application of this platform to HTP formulation screening.

### 3.3. Establishment of Robust HTP Analytics for Formulation Stability Assessment

Accurate and rapid assessment of stability-indicating CQAs of AAV is essential for HTP formulation screening. We evaluated the feasibility of applying Stunner as an HTP analytics platform for characterizing critical AAV stability-indicating CQAs such as vector genome titer and particle size [39]. The performance of Stunner was further evaluated against conventional manual analytical methods for AAV characterization such as vector genome titer by ddPCR and particle size by DLS. The goal is to verify if comparable results could be achieved between using the HTP analytics platform and the conventional manual methods.

**Figure 4:**
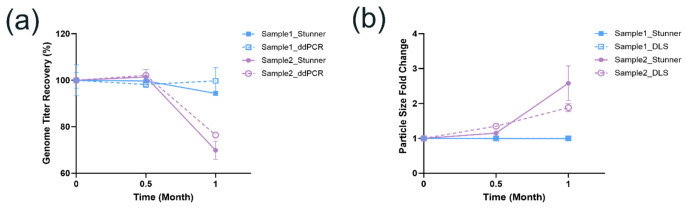
Evaluation of performance of Stunner for automated multi-attribute analysis. Two distinct AAV1 formulations at 1.2 × 10^14^ vg/mL were evaluated over one month at 5 °C. (**a**) Percent vector genome titer recovery was measured using Stunner (n = 3) and droplet digital PCR (ddPCR) (n = 4); (**b**) particle size fold changes were determined using Stunner (n = 3) and dynamic light scattering (DLS) (n = 5). Similar stability trends were observed using Stunner and manual analytical methods.

In this study, two AAV1 formulations at approximately 1.2 × 10^14^ vg/mL but with different excipient compositions were incubated at 5 °C for up to one month. Then, the vector genome titer and particle size of the samples were analyzed using Stunner and the conventional methods (i.e., ddPCR and DLS). As shown in Figure 4a,b, minor changes in vector genome titer and particle size were observed for Sample 1 following one month’s incubation at 5 °C, indicating minimal degradation. However, Sample 2 exhibited significant degradation, evidenced by dramatically reduced vector genome titer recovery and increased particle size, especially after one month’s incubation at 5 °C. Importantly, despite small differences in the absolute values reported using Stunner and the conventional methods as well as increased variability commonly associated with heavily degraded samples, Stunner consistently identified the same stability trends with comparable precision as conventional manual methods [39]. The results from this study demonstrate Stunner is a reliable AAV characterization tool for formulation stability assessment. With significantly improved throughput, this robust HTP analytics platform can streamline screening and optimization of AAV formulations, accelerating development timelines while maintaining similar analytical rigor achieved with conventional AAV characterization methods.

### 3.4. A Case Study for High-Titer AAV1 Formulation Screening

To assess the capability of this automated HTP platform for AAV formulation screening, we conducted a systematic formulation screening study using AAV1 as the model vector.

#### 3.4.1. Design Formulation Matrix for HTP Screening

**Figure 5:**
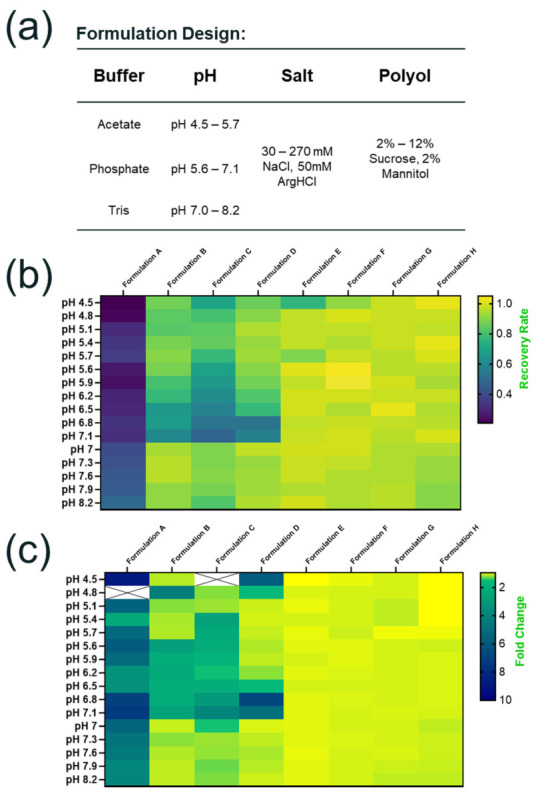
Case study: formulation design and automated high-throughput (HTP) screening by assessing stability-indicating quality attributes. (**a**) Formulation design for evaluating three buffer types and broad range of key formulation excipients. The heatmap shows formulation stability assessment results as a function of pH and excipient combinations (n = 2); the vector genome titer recovery (**b**) and fold change of particle size (**c**) were calculated by comparing the results from samples before and after one month’s incubation at 5 °C. The “X” symbol indicates a more than 10-fold increase in particle size.

We designed a large formulation matrix exploring 128 distinct conditions across multiple buffer systems, pH ranges, and excipient combinations (Figure 5a). Three buffer systems were evaluated, and each tested over a wide pH range with a pH interval of 0.3 units. Specifically, acetate buffers were assessed for the pH range from 4.5 to 5.7, phosphate buffers from 5.6 to 7.1, and tris buffers from 7.0 to 8.2. Salt is a critical excipient for maintaining AAV stability, and NaCl is the most commonly used salt type in AAV formulations. To explore the formulation design space for salt and ionic strength, we varied the NaCl concentrations from 30 mM to 270 mM. Additionally, 50 mM ArgHCl was included in some formulations to assess the impact of salt type as well as additional stabilizer on AAV stability. Sucrose, with a known effect on enhancing the freeze/thaw and thermal stability of AAV, was assessed at concentrations from 2% to 12%. Mannitol has been included in previously published lyophilized AAV formulations mainly as a bulking agent [40,41]. Whether it has a stabilizing effect in AAV liquid formulations remains to be evaluated. Thus, a fixed concentration of mannitol at 2% was evaluated in this study to simplify the formulation design. Unless otherwise specified, all formulations were supplemented with 0.005% *w/v* P188.

#### 3.4.2. Identify Optimal AAV1 Formulations Using HTP Platform

The AAV1 stock material was concentrated to 6 × 10^14^ vg/mL immediately before 10-fold dilution into a 96-well plate during the automated compounding process, yielding final AAV1 titer at approximately 6 × 10^13^ vg/mL across all formulation conditions. The plate was then sealed following the sealing protocol described in Section 2.2 and stored in a 5 °C refrigerator for up to one month.

Upon completion of one month’s incubation, the plate was removed from the refrigerator and transferred to the Stunner plate for measurement of vector genome titer and particle size. The data were summarized and presented in a heat map format. As shown in Figure 5b, changes in AAV1 stability were assessed by calculating the percentage recovery of vector genome titer relative to that of the T_0_ sample. A higher recovery rate indicates less loss of titer and improved formulation stability. Aggregation, another major degradation pathway for AAV, was assessed through increases in particle size relative to that of the T_0_ sample (Figure 5c). A value of fold of increase close to 1.0 indicates AAV remains stable in the formulation with minimal aggregation observed.

The results from this HTP formulation screening study, as shown in Figure 5b,c, revealed striking patterns across the formulation space and clearly demonstrated the capability of this platform screening for differentiating large formulation conditions with diverse stabilizing effects. Based on an assessment of the changes in vector genome titer and particle size under 5 °C up to one month, we defined the approach of scoring for identifying optimal formulation candidates for further validation. For example, formulations were considered “hit” when they demonstrated ≥99.5% recovery of vector genome titer and ≤1.02-fold of particle size increase after one-month storage at 5 °C. These threshold values were established empirically, balancing the overall performance of all the candidate formulation conditions screened and the desired number of “hits” to be selected for comprehensive stability confirmation studies. When formulations with highly similar formulation parameters such as pH and excipient concentration demonstrated comparable stability profiles, only one representative formulation candidate was selected to avoid redundancy in validation studies. Overall, four top-performing formulation candidates were selected as the potential “hit” and advanced to the subsequent validation studies.

#### 3.4.3. Validate the “Hit” AAV1 Formulations Using Stability Confirmation Studies

The stability of the four top-performing “hit” AAV1 formulations identified from the HTP screening study were subsequently validated with a traditional formulation stability confirmation study by filling the formulations into standard CZ vials. To provide an objective evaluation of the performance of our identified formulation hits, we incorporated a reference formulation (F0) in the validation study, namely phosphate-buffered saline (PBS) buffer with 0.005% *w/v* P188, as it is often used in research and clinical development for AAV [8,42]. In this study, 0.4 mL of each of the five formulations with AAV1 vector genome titer at 1.2 × 10^14^ vg/mL was filled into a 2 mL CZ cyclic olefin polymer (COP) vial and subsequently sealed with a rubber stopper and an aluminum seal to maintain container closure integrity. Since AAVs tend to show an increased propensity to aggregate at higher titers, we chose a much higher vector genome titer during the validation study than what was used in the HTP screening study (i.e., 1.2 × 10^14^ vs. 6 × 10^13^ vg/mL) to facilitate identification of any potential stability differences among these formulation conditions. The performance of these formulations was initially evaluated under 5 °C for up to one month by assessing changes in vector genome titer by ddPCR and particle size by DLS. The “hit” formulations which demonstrated satisfactory stability at 5 °C during initial assessment were further evaluated under additional stress conditions including incubation at 25 °C for one month, two cycles of freeze/thaw, or agitation by vortexing for up to two hours. These stress conditions were selected to simulate the conditions that the AAV formulation may be subjected to during clinical manufacturing, shipping and handling, as well as clinical use.

**Figure 6:**
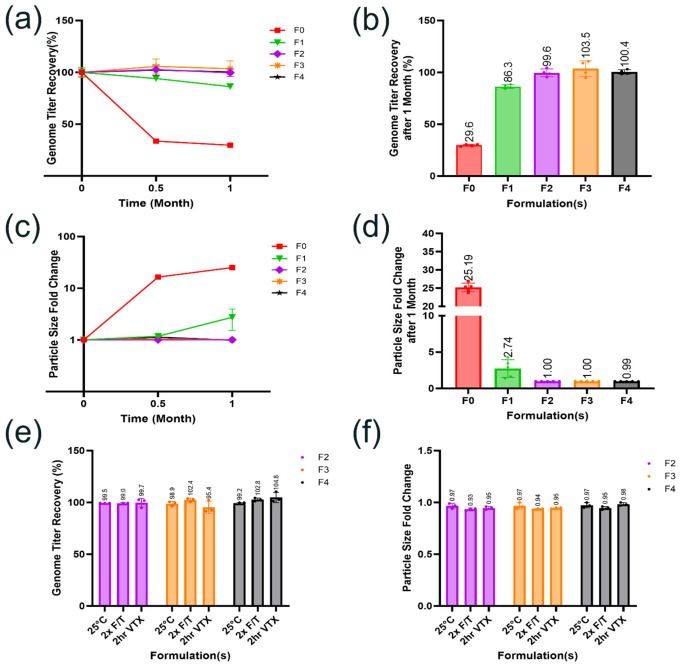
Validation of the “hit” AAV1 formulations using stability confirmation study. Five AAV1 formulations at ~1.2 × 10^14^ vg/mL including F0 and 4 “hit” formulations (F1–F4) were assessed. Analysis of vector genome titer recovery (**a**,**b**) and fold change of particle size (**c**,**d**) at 5 °C for one month identified 3 top-performing “hit” formulations (F2–F4). These 3 formulations were further evaluated for the two stability-indicating quality attributes under additional stress conditions (**e**,**f**) and demonstrated satisfactory stability under all conditions evaluated. 2x F/T: two freeze/thaw cycles; 2 hr VTX: vortexing agitation for two hours. n = 3 or 4 for each sample. All samples were equilibrated to room temperature for a minimum of 30 min prior to analytical measurements.

Figure 6a–d summarize the results of the initial stability assessment study for the five AAV1 formulations, including the reference formulation F0 and the four “hit” formulations (F1 to F4). Notably, formulations F2, F3, and F4 exhibited good stability. After one month at 5 °C, all three formulations demonstrated within 4% changes in vector genome titer recovery compared to the T0 sample. Also, no detectable increase in particle size was observed in these formulations. In contrast, AAV1 in the PBS formulation (F0) showed substantial degradation with extended incubation at 5 °C. After one month at 5 °C, approximately 70% vector genome titer losses along with a 25-fold increase in particle size were observed, which is likely due to significant aggregation. Formulation F1, although it showed much improved performance compared to F0, approximately 14% vector genome titer losses along with a 2.7-fold increase in particle size were observed after one month at 5 °C, indicating stability issues with F1. Thus, only F2, F3, and F4 were advanced to the next stability assessment study. As shown in Figure 6e,f, all these three formulations achieved within 5% changes in vector genome titer recovery and a minimal increase in particle size compared to the T0 sample under the stress conditions evaluated, including after incubation at 25 °C for one month, two freeze/thaw cycles or vortexing for two hours. These results confirmed that the three “hit” formulations F2, F3, and F4 identified from the HTP screening demonstrated satisfactory stability as an AAV1 drug product formulation. Furthermore, the case study validates this HTP screening platform’s capacity to rapidly identify optimal formulation conditions from a diverse design space while consuming minimal vector material.

## 4. Discussion

### 4.1. Critical Considerations for HTP Platform

The workflow for the automation-assisted HTP formulation screening platform, along with any optimizations performed in this study, are typically part of the evaluation or testing phase in a design, build, test, learn (DBTL) cycle [43,44]. To successfully apply this platform, it is essential to adjust and train the system before conducting the formulation screening study. For instance, we fine-tuned the liquid class settings to ensure the system accurately pipettes the correct amount of stock solution into the target plate. Detailed adjustments, such as tip mixing, sample addition processes, dead volume control, and the choice of stamp head or column channel pipetting, are critical factors to evaluate during platform development. These factors can significantly impact the results of the formulation screening study and must be meticulously optimized. Given the potential instability concern for concentrated AAV stock solutions, they must be prepared immediately prior to the AAV compounding step to preserve the quality and stability of the AAV samples. Proper sealing of the plates is also essential to prevent evaporation, which can adversely affect the integrity of the samples and introduce uncontrolled variability across the 96-well plate. The plates must be sealed immediately after completion of the formulation compounding step to maintain a controlled environment during the formulation stability assessment study.

### 4.2. Advantages

There are several advantages of an automation-assisted HTP platform for AAV formulation screening. Firstly, the HTP platform enables rapid and efficient preparation of formulation buffers and AAV compounding, significantly reducing the time and labor required for these processes. For instance, it takes one analyst about 40 min to prepare 96 different formulations and 20 min to complete AAV compounding on a 96-well plate. Thus, it takes merely about one hour to have 96 AAV formulations ready for screening. In contrast, a similar amount of work could take about one week for an analyst to accomplish when prepared manually. More importantly, the significantly improved efficiency and productivity offered by the HTP platform makes it feasible to explore a much larger formulation design space than what can be achieved with a traditional manual formulation screening study, increasing the possibility for identifying optimal formulations. This is crucial for addressing the growing demand for developing robust AAV formulations for the increasing number of AAV serotypes and engineered capsids. Secondly, by reducing human intervention, the platform minimizes the possibility of human error as well as variability commonly associated with manual sample handling, especially when preparing a large quantity of formulations for screening study.

### 4.3. Limitations

Despite its advantages, the automation-assisted HTP platform for AAV formulation screening has certain limitations. One primary limitation is the requirement for specialized equipment and infrastructure, such as the Hamilton Microlab STAR and sophisticated HTP analytical characterization instruments, which may pose challenges for resource-limited laboratories. Additionally, while the platform reduces time and labor, the workflow is not fully automated and still demands expertise in manual preparing concentrated AAV stock solutions, handling characterization instruments, and performing data analysis to ensure reliable results. The initial setup phase can be time-consuming, requiring systematic adjustments and process optimization.

### 4.4. Future Directions

We anticipate implementation of a closed-loop DBTL cycle would enable transition from traditional empirical approaches to data-driven formulation discovery, accelerating the formulation development [45,46,47]. This can be achieved by integrating artificial intelligence (AI) and machine learning (ML) with the HTP automation platform. ML models trained on comprehensive formulation datasets could identify non-obvious correlations between formulation parameters and product stability. Taking advantage of the large datasets from the HTP screening, predictive algorithms could also be developed and help to effectively narrow the vast formulation design space, focusing resources on promising candidates. These AI and ML-enhanced approaches have the potential to substantially reduce development timelines for stable AAV formulations across multiple serotypes and engineered capsids, accelerating translation of gene therapy products to clinical applications. Also, with the fast advancement in AAV analytical technology, additional HTP analytics for probing the conformational and/or colloidal stability of AAVs could be evaluated for feasibility to be incorporated into this HTP automation platform, which should be highly helpful in supporting AAV formulation screening studies.

## 5. Conclusions

We successfully established a HTP platform utilizing a 96-well plate format that enables rapid, resource-efficient screening of diverse formulation conditions with minimal material consumption. The automated system achieved exceptional precision across critical performance parameters, including pH control, excipient concentration accuracy, and AAV compounding reproducibility. Our innovative multi-layer sealing strategy effectively eliminated evaporation concerns and edge effects during extended incubation periods, ensuring data consistency across all plate positions. By leveraging this platform, we screened 128 distinct formulation conditions for AAV1 at 6 × 10^13^ vg/mL. We systematically evaluated the influence of key formulation variables including pH, ionic strength, and stabilizing excipients. Taking advantage of advanced HTP analytical techniques, we achieved simultaneous assessment of AAV stability through multiple CQAs, allowing identification of optimal formulation regions within the design space. The most promising formulations were subsequently validated at a high AAV titer of 1.2 × 10^14^ vg/mL under conditions simulating pharmaceutical manufacturing, storage, and clinical use. These validation studies confirmed that formulations identified through our HTP approach maintain exceptional stability during extended refrigerated storage, room temperature exposure, freeze/thaw cycling, and agitation stress conditions, with performance comparable to a commercially approved reference formulation for long-term refrigerated storage of AAV.

In summary, this work demonstrates the promising ability to apply automation-assisted approaches for accelerating the AAV formulation development timelines while enhancing the probability of identifying optimal conditions, especially for molecules with stability challenges. This approach can support the development of both early-stage “platform” formulations for expedited clinical entry and robust formulations for commercial products. The platform established here represents a valuable addition to the gene therapy development toolkit, addressing a critical bottleneck in the advancement of these transformative therapeutic modalities.

## Data Availability

The original contributions presented in this study are included in the article/Supplementary Material. Further inquiries can be directed to the corresponding authors.

## Supplementary Materials

The following supporting information can be downloaded at https://www.mdpi.com/article/10.3390/pharmaceutics18050608/s1.

## Abbreviations

The following abbreviations are used in this manuscript:

AAV	Adeno-associated virus
vg	Vector genome
HTP	High-throughput
DLS	Dynamic light scattering
LC	Liquid chromatography
CQAs	Critical quality attributes
P188	Poloxamer 188
ArgHCl	Arginine hydrochloride
NaCl	Sodium chloride
MWCO	Molecular weight cutoff
CZ	Crystal Zenith^®^
SLS	Static light scattering
ssDNA	Single-stranded DNA
ddPCR	Droplet digital PCR
CV	Coefficient of variation
RP-UPLC	Reversed-phase ultra-high-performance liquid chromatography
SEC-CAD	Size exclusion chromatography with charged aerosol detector
SEC-MALS	Size exclusion chromatography with multi-angle light scattering
PBS	Phosphate-buffered saline
COP	Cyclic olefin polymer
DBTL	Design, build, test, learn
AI	Artificial intelligence
ML	Machine learning
TOST	Two one-sided tests
