# Supplementary material for: A High-Throughput Automation Platform for Accelerated AAV Stability Optimization
Source: Pharmaceutics. 2026 May 16;18(5):608. doi: 10.3390/pharmaceutics18050608 (PMC13211149; doi:10.3390/pharmaceutics18050608)
Supplement: Supplementary file 1 [file pharmaceutics-18-00608-s001.zip › Supplemental Document 2.docx]

A High-Throughput Automation Platform for Accelerated AAV Stability Optimization

Shuai Li, Xiaoyan Wang, Li Zhi^#^, Mohammed Shameem, Dingjiang Liu^#^

Drug Product Development and Technology, Regeneron Pharmaceuticals Inc., Tarrytown NY 10591

Corresponding author:

[li.zhi@regeneron.com](mailto:li.zhi@regeneron.com)

[dingjiang.liu@regeneron.com](mailto:dingjiang.liu@regeneron.com)

**Supplemental Figures**


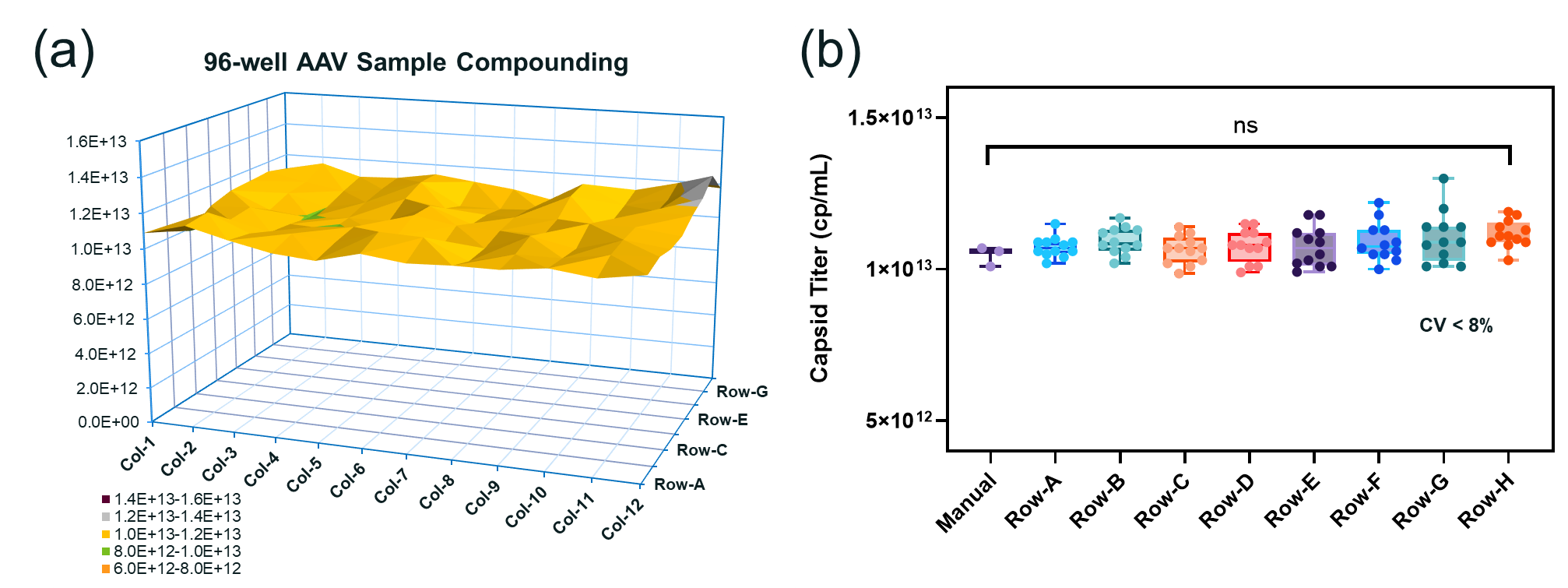


**Figure S1.** Validation of automated recombinant adeno-associated virus (AAV) compounding process using size exclusion chromatography with multi-angle light (SEC-MALS) method. (a) Capsid titer (cp/mL) distribution across a 96-well plate prepared by diluting AAV concentrate to a target titer of 1.1E+13 cp/mL. (b) Analysis of consistency in AAV titer after compounding across the 8 rows A-H showed <8% coefficient of variation (CV) (n=12 wells per row). Statistical comparison (one-way ANOVA) between manually prepared samples (n=3) and each row of automated prepared samples showed no significant differences (p > 0.05). The results provided orthogonal validation data to the vector genome (vg) titer results presented in Figure 3, confirming the robustness of the automated compounding process.


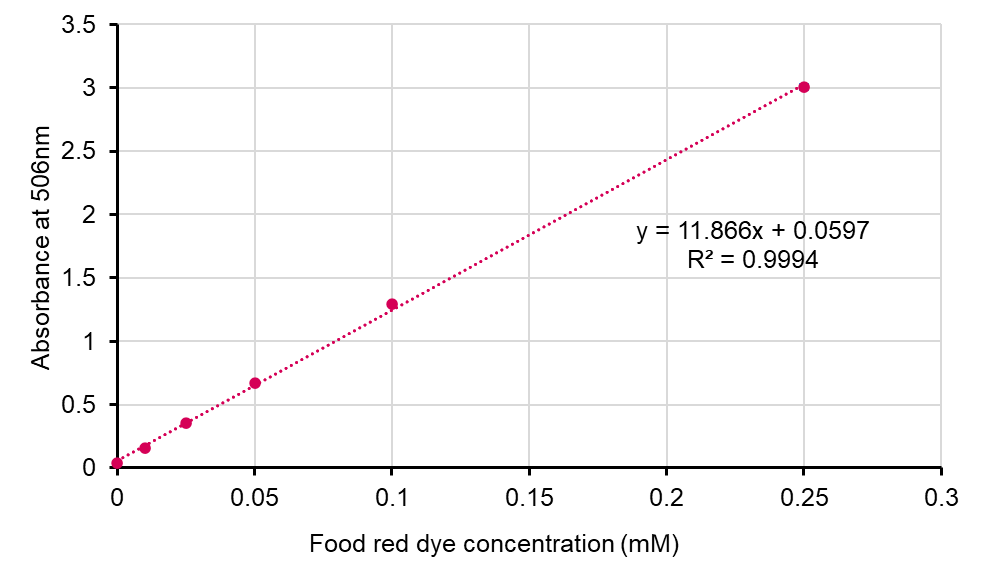


**Figure S2.** Food red dye absorbance linearity range test. The absorbance of 200 µL of red food dye with serial concentration (0 to 0.25 mM) was measured at 506 nm (A506) using SpectraMax 190 Microplate Reader. (n=3 for each concentration)
